# 100. Safety Analysis of Live-Attenuated Measles, Mumps, Rubella Vaccine Among Hematopoietic Cell Transplant Recipients Vaccinated Within Two Years of Transplant

**DOI:** 10.1093/ofid/ofab466.100

**Published:** 2021-12-04

**Authors:** Xhoi Mitre, Monica Feeley, Amy C Sherman, Stephen R Walsh, Matthew Cheng, Sanjat Kanjilal, Vincent T Ho, Lindsey R Baden, Nicolas C Issa, Michaël Desjardins

**Affiliations:** 1 Brigham and Women's Hospital, Boston, Massachusetts; 2 Harvard Medical School/Brigham and Women's Hospital, Boston, Massachusetts; 3 Brigham & Women's Hospital, Boston, Massachusetts; 4 McGill University Health Centre, Montreal, Quebec, Canada; 5 Harvard Medical School and Harvard Pilgrim Healthcare Institute, Jamaica Plain, MA; 6 Dana-Farber Cancer Institute, Boston, Massachusetts

## Abstract

**Background:**

Measles, mumps and rubella (MMR) vaccine is a live-attenuated vaccine usually contraindicated within the first two years of hematopoietic cell transplant (HCT). During the 2019 measles outbreak at our center, the benefits of administering MMR vaccine within the first two years after HCT were weighed against the potential risks.

**Methods:**

We conducted a retrospective review of patients who received MMR vaccination within two years of an autologous or allogeneic HCT. Patients’ demographics, date and type of HCT, underlying hematologic disease, type of immunosuppressive therapy and date of MMR vaccination were extracted from the electronic medical record. Adverse reactions that could be related to the vaccine were collected for up to 42 days post-vaccination and all hospitalizations and deaths following vaccination were reviewed.

**Results:**

A total of 129 patients (75 autologous and 54 allogeneic HCT) were vaccinated between 300-729 days after HCT (median of 718 days). The median age at vaccination was 61 years old, 57% of the patients were male and 43% were on immunosuppressive therapy, 87% of whom were on maintenance therapy for multiple myeloma after auto-HCT. Seven patients (5%) had adverse reactions within 42 days of vaccination: six had respiratory tract infections (three with associated fever) and one had a rash leading to a brief hospitalization. This was a 37-year-old female who had an allogeneic HCT 542 days prior to MMR vaccination. She presented with a centrifugal maculopapular rash that was confirmed to be caused by the vaccine strain rubella virus (Fig 1). She fully recovered without sequalae. There was no other vaccine-associated illness identified in the cohort, after a median follow-up of 676 days.

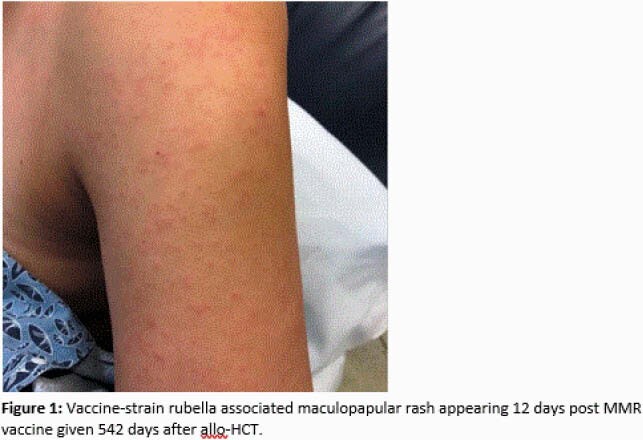

**Conclusion:**

MMR vaccine appears to be well tolerated in selected HCT recipients when given earlier than 2 years after transplant. No attributable severe outcomes or deaths were described. A mild uncomplicated case of vaccine-associated rubella illness was seen after vaccination. In the setting of a measles outbreak, assessment of potential risks and benefits of MMR vaccination given within two years of HCT remains important.

**Disclosures:**

**Stephen R. Walsh, MDCM**, **Janssen Vaccines** (Scientific Research Study Investigator)**Regeneron** (Scientific Research Study Investigator)**Sanofi Pasteur** (Scientific Research Study Investigator) **Matthew Cheng, MD**, **GEn1E Lifesciences** (Advisor or Review Panel member)**Kanvas Biosciences** (Board Member, Shareholder)**nplex biosciences** (Advisor or Review Panel member) **Sanjat Kanjilal, MD, MPH**, **GlaskoSmithKline** (Advisor or Review Panel member) **Nicolas C. Issa, MD**, **AiCuris** (Scientific Research Study Investigator)**Astellas** (Scientific Research Study Investigator)**GSK** (Scientific Research Study Investigator)**Merck** (Scientific Research Study Investigator)

